# Supplementary material for: Safeguarding against external intrusions utilizing adaptive bio-inspired multi-population anomaly detection for IoT network
Source: PLoS One. 2026 Mar 27;21(3):e0344685. doi: 10.1371/journal.pone.0344685 (PMC13028378; doi:10.1371/journal.pone.0344685)
Supplement: Attachment — Submitted filename: Plosone response.pdf [file pone.0344685.s003.pdf]

Response to Reviewer Comments

We thank the Editor for handling the review process and the anonymous reviewers for their valuable comments and suggestions. In the revised manuscript, we have thoroughly addressed all the comments of the Editor, whose details are as follows.

Response to Reviewer 1

Comments to the Author: The revised manuscript shows clear and commendable improvement over the original submission. The authors have systematically addressed all prior reviewer comments with well-documented changes:

A new novelty section was added (Section 1.1), effectively highlighting the contribution of CMGODE.

The literature review now includes recent work on deep learning-based IDS systems.

The methodology and algorithm (chaotic multi-population GOA with DE) are thoroughly explained with mathematical detail, parameter settings, and motivation for design choices (e.g., use of the logistic map, sub-populations, DE steps).

Statistical tests (Friedman test, Holm, and Li post hoc tests) are included and interpreted properly.

The manuscript also now discusses model generalization, real-time feasibility, adversarial robustness, and false alarm rates — all critical for intrusion detection in IoT environments.

Response tables and revised figures have been improved in structure and clarity.

Authors' Response: *We are thankful to the reviewer for considering all comments and recommended to acceptance with minor revisions.*

Comments to the Author: Minor issues remain only at the language level. A light editorial review for grammar and phrasing will enhance the manuscript's clarity.

Author's Response: *Sincerely thankful for accepting our manuscript. We apologize for the lack of readability of the manuscript; in the previous version of the*

manuscript, we tried to improve the linguistic quality of the manuscript, but we did not meet your expectations. We have rechecked the revised manuscript by a native English speaker from the abstract to the conclusion section. Several minor grammatical and spelling edits have been made throughout the text that have improved linguistic quality as well as checked the numbering in references. We hope that this revised manuscript meets your expectations.

Comments to the Author: Additionally, please add explicit links to the public datasets (BoT-IoT and UNSW-NB15) in the Data Availability Statement for full transparency.

Author Response: *We apologize for the lack of explicit links of the public datasets (BoT-IoT and UNSW-NB15) in previous manuscript. In response, we have included links of the public datasets (BoT-IoT and UNSW-NB15) in the Data Availability Statement.*
