# Supplementary material for: Safeguarding against external intrusions utilizing adaptive bio-inspired multi-population anomaly detection for IoT network
Source: PLoS One. 2026 Mar 27;21(3):e0344685. doi: 10.1371/journal.pone.0344685 (PMC13028378; doi:10.1371/journal.pone.0344685)
Supplement: Attachment — Submitted filename: response letter.pdf [file pone.0344685.s004.pdf]

Response to Reviewer Comments

We thank the Editor for handling the review process and the anonymous reviewers for their valuable comments and suggestions. In the revised manuscript, we have thoroughly addressed all the comments of the Editor, whose details are as follows.

Response to Reviewer 1

Comments to the Author: The revised manuscript demonstrates a substantial and commendable improvement over the original submission. The authors have carefully addressed prior reviewer concerns and significantly strengthened the manuscript in several key areas: Novelty: Section 1.1 now clearly articulates the novelty of the CMGODE algorithm, justifying the use of chaos theory and multi-population strategies in the context of intrusion detection. Methodology: The proposed algorithm is thoroughly explained with mathematical formulations, flowcharts (see Figures 1 and 2, pages 16 and 22), and pseudocode, enhancing reproducibility. Experimental Rigor: Use of BoT-IoT and UNSW-NB15 datasets, comprehensive pre-processing, and comparison against multiple algorithms strengthen the empirical foundation. Statistical Validation: Inclusion of statistical hypothesis testing (e.g., Friedman test) supports the significance of the findings. Practical Considerations: Discussion on generalization, adversarial robustness, false alarm rates, and real-time feasibility is a major strength.

Authors' Response: *We are thankful to the reviewer for considering all comments and recommended to acceptance with minor revisions.*

Comments to the Author: Ensure the manuscript undergoes final copyediting for minor language issues. Consider summarizing key experimental findings in a visual table in the conclusion section for quicker reference by readers.

Once these minor editorial issues are addressed, I believe this manuscript is suitable for publication.

Author's Response: *Sincerely thankful for accepting our manuscript. We apologize for the lack of readability of the manuscript; in the previous version of the manuscript, we tried to improve the linguistic quality of the manuscript, but we did*

not meet your expectations. We have rechecked the revised manuscript by a native English speaker from the abstract to the conclusion section. Several minor grammatical and spelling edits have been made throughout the text that have improved linguistic quality as well as checked the numbering in references. Furthermore, in revised manuscript, we have summarized key experimental findings in the conclusion section for better understanding.

We hope that this revised manuscript meets your expectations.
